# Low birthweight is associated with a higher incidence of type 2 diabetes over two decades independent of adult BMI and genetic predisposition

**DOI:** 10.1007/s00125-023-05937-0

**Published:** 2023-06-12

**Authors:** Rasmus Wibaek, Gregers S. Andersen, Allan Linneberg, Torben Hansen, Niels Grarup, Anne Cathrine B. Thuesen, Rasmus T. Jensen, Jonathan C. K. Wells, Kasper A. Pilgaard, Charlotte Brøns, Dorte Vistisen, Allan A. Vaag

**Affiliations:** 1 Clinical Research, Steno Diabetes Center Copenhagen, Herlev, Denmark; 2 Center for Clinical Research and Prevention, Copenhagen University Hospital - Bispebjerg and Frederiksberg, Copenhagen, Denmark; 3 Department of Clinical Medicine, Faculty of Health and Medical Sciences, University of Copenhagen, Copenhagen, Denmark; 4 Novo Nordisk Foundation Center for Basic Metabolic Research, Faculty of Health and Medical Sciences, University of Copenhagen, Copenhagen, Denmark; 5 Childhood Nutrition Research Centre, Population Policy and Practice Research and Teaching Department, UCL Great Ormond Street Institute of Child Health, London, UK; 6 Department of Public Health, University of Copenhagen, Copenhagen, Denmark; 7 Lund University Diabetes Centre, Lund University, Malmö, Sweden; 8 Department of Endocrinology, Skåne University Hospital, Malmö, Sweden

**Keywords:** Birthweight, Cohort, Developmental origins, Fetal programming, Genetic susceptibility, Low birthweight, Type 2 diabetes

## Abstract

**Aims/hypothesis:**

Low birthweight is a risk factor for type 2 diabetes. Most previous studies are based on cross-sectional prevalence data, not designed to study the timing of onset of type 2 diabetes in relation to birthweight. We aimed to examine associations of birthweight with age-specific incidence rate of type 2 diabetes in middle-aged to older adults over two decades.

**Methods:**

Adults aged 30–60 years enrolled in the Danish Inter99 cohort in 1999–2001 (baseline examination), with information on birthweight from original birth records from 1939–1971 and without diabetes at baseline, were eligible. Birth records were linked with individual-level data on age at diabetes diagnosis and key covariates. Incidence rates of type 2 diabetes as a function of age, sex and birthweight were modelled using Poisson regression, adjusting for prematurity status at birth, parity, polygenic scores for birthweight and type 2 diabetes, maternal and paternal diabetes history, socioeconomic status and adult BMI.

**Results:**

In 4590 participants there were 492 incident type 2 diabetes cases during a mean follow-up of 19 years. Type 2 diabetes incidence rate increased with age, was higher in male participants, and decreased with increasing birthweight (incidence rate ratio [95% CI per 1 kg increase in birthweight] 0.60 [0.48, 0.75]). The inverse association of birthweight with type 2 diabetes incidence was statistically significant across all models and in sensitivity analysis.

**Conclusions/interpretation:**

A lower birthweight was associated with increased risk of developing type 2 diabetes independent of adult BMI and genetic risk of type 2 diabetes and birthweight.

**Graphical Abstract:**

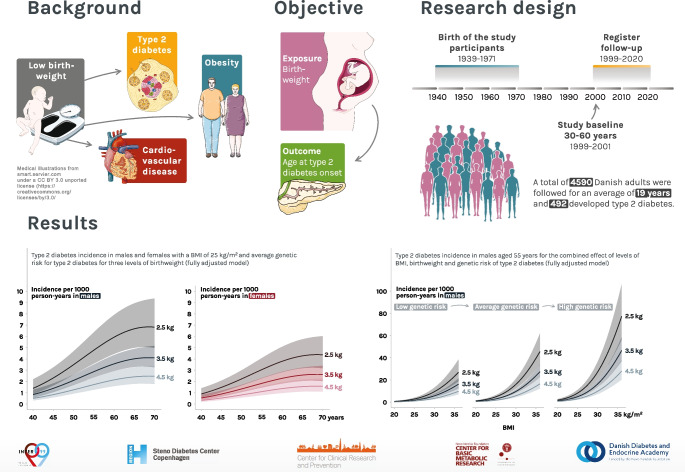

**Supplementary Information:**

The online version contains supplementary material available at 10.1007/s00125-023-05937-0.


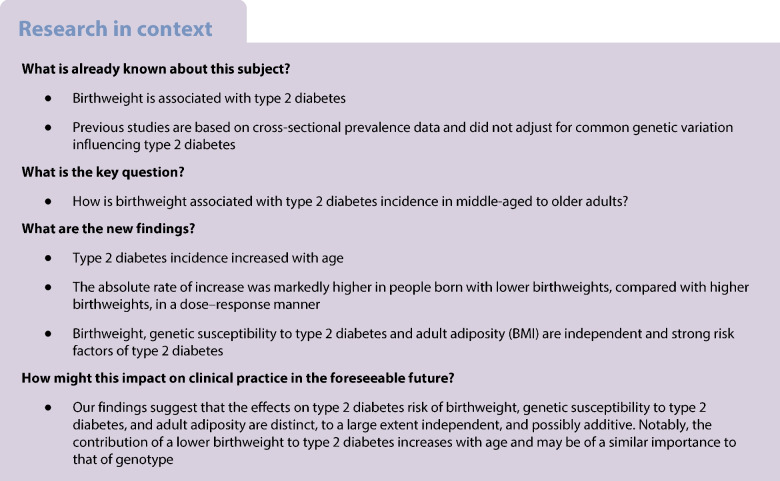


## Introduction

Type 2 diabetes, affecting more than 400 million people globally, represents one of the most significant global health challenges of our time [1]. The risk of type 2 diabetes is affected not only by genetic susceptibility and lifestyle factors but also by an adverse intrauterine environment associated with low birthweight (LBW). A large systematic review including 31 populations and 152,084 individuals found that a 1 kg increase in birthweight was associated with 20% reduced risk of type 2 diabetes [2] and LBW has consistently been found to be inversely associated with a more adverse glucometabolic profile [3]. Additionally, several studies have also found high birthweight to increase the adult risk of type 2 diabetes, suggesting an increased risk at both ends of the birthweight spectrum [4]. The Developmental Origins of Health and Disease (DOHaD) hypothesis proposes that insults during critical windows of developmental plasticity [5], such as inadequate nutrition in the developing fetus, affect fetal growth and induce permanent changes to the structure and function of organs and tissues, increasing the risk of developing obesity, type 2 diabetes and cardiovascular disease in adulthood [6, 7]. Data from historical humanitarian famine catastrophes in Ukraine and the Netherlands have supported the initial reports from the UK [8] of an adverse intrauterine environment associated with LBW playing a significant role in type 2 diabetes risk [9, 10]. This has subsequently been confirmed and validated in meta-analyses and examined mechanistically in human and animal studies [2-4, 11-13]. Based on cross-sectional prevalence data in the Danish Inter99 cohort, we previously found an inverse association of birthweight with type 2 diabetes risk in people aged 30–60 years [14]. Similarly, most previous studies were based on prevalence data in elderly adults to estimate type 2 diabetes risk and therefore were not designed to study the timing of onset of type 2 diabetes in relation to birthweight. We aimed to examine associations of birthweight with age- and sex-specific incidence rates of type 2 diabetes and assess how adult BMI and genetic susceptibility of type 2 diabetes may modify these associations.

## Methods

### Study design and setting

**Fig. 1:**
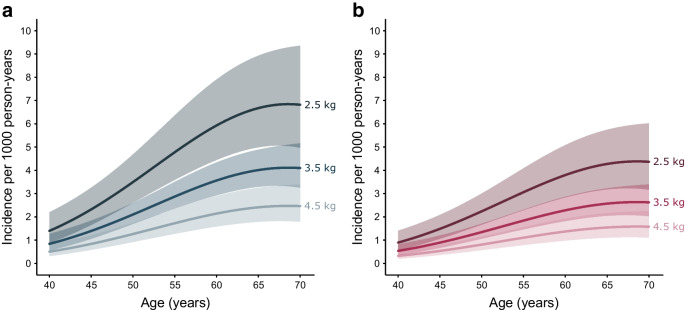
Incidence of type 2 diabetes per 1000 PY as a function of age, birthweight, sex, prematurity status, parity, PS for birthweight and type 2 diabetes, maternal and paternal history of diabetes, socioeconomic status and adult BMI (*n*=4102). The solid lines and shaded areas show the estimated incidence rates and 95% CIs, respectively, for male (**a**) and female (**b**) participants aged 40–70 years, with birthweight levels of 2.5, 3.5 and 4.5 kg, who were firstborns, who were term births, and who had a study population median PS for birthweight and type 2 diabetes, no maternal and paternal history of diabetes, a high socioeconomic status (i.e. currently working with >1 year of education [referring to vocational, professional or academic adult education beyond primary or secondary school]) and BMI of 25 kg/m^2^. Age and BMI are specified as second-degree polynomials in the model

A total of 6784 adults aged 30–60 years at the baseline examination were enrolled in the Danish Inter99 cohort from 1999 to 2001 (electronic supplementary material [ESM] Fig. 1) [15]. Study participants with a registered diagnosis of either type 1 or 2 diabetes at baseline examination (*n*=118) or with no birth record information (*n*=1992) were excluded [14]. Furthermore, multiple pregnancies (*n*=84) were excluded to remove the influence of the different intrauterine environment in multiple compared with single pregnancies. This resulted in a final study population of 4590.

Detailed ethnicity and race information was not captured for the Inter99 population at the study baseline in 1999. However, self-reported nationality was Danish in 95% of the participants, and thus this appears to be a homogeneous (primarily European white) population.

### Data collection

#### Midwife records

Information on sex, date of birth, prematurity status, parity and objectively measured weight at birth was available from original midwife records from the Danish State Archives from 1939–1971 as previously described [16]. A child was categorised as either term (36–41 completed weeks of gestation) or preterm. The prematurity status was assessed by the midwife based on the time since the first day of the last menstrual period as well as clinical signs of prematurity.

#### Type 2 diabetes register

Midwife records were linked with individual-level data from Danish registers through the unique personal identification number in the Danish civil registration system. Assessment of type 2 diabetes status and date of diagnosis was based on a newly established Danish Diabetes Register (DMreg). A detailed description of this register is provided in the ESM.

### OGTT

A 75 g OGTT was administered to participants without known diabetes at the baseline examination [15]. Venous blood samples were drawn in the fasting state before glucose administration, and again at 30 and 120 min. Concentrations of plasma glucose at 0, 30 and 120 min were analysed using the hexokinase/G6P-DH method (Boehringer Mannheim, Mannheim, Germany). DNA was isolated and kept at −80°C.

### Genotyping and genetic risk score computation

Genotyping was carried out using Illumina OmniExpress-24 Bead Chip (Illumina, San Diego, CA, USA). Genotypes were called using GenCall in GenomeStudio (v2011.1; Illumina) and variants were included if the call rate was ≥98% and Hardy Weinberg equilibrium *p*>1×10^−5^. Data were phased using Eagle and Shapeit and imputed using Phase 3 1000G and HRC panel using the Michigan imputation server. Samples with mismatch between genetic and phenotypic sex, ethnic outliers or call rate <95% were excluded. Two weighted polygenic scores (PS) were calculated: (1) for type 2 diabetes, using a published score consisting of 6.9 M SNPs on HRC imputation [17]; and (2) for birthweight, using summary statistics for 146 SNPs that were genome-wide significantly associated with birthweight data from the Early Growth Genetics consortium [18] on 1000G imputation, as previously described [19]. For the type 2 diabetes PS, 94% SNPs were included, and all SNPs were included in the birthweight PS.

### Covariates

Information on maternal and paternal history of diabetes and socioeconomic status was obtained from questionnaires at the Inter99 baseline examination. Weight and height were objectively measured to the nearest 0.1 kg and 1 cm, respectively, at the baseline examination. BMI was calculated as weight in kg divided by height squared in meters (kg/m^2^).

### Statistical methods

An individual’s risk time began at inclusion in the baseline examination in 1999–2001 and ended at time of diabetes diagnosis (type 1 or 2), emigration from Denmark, death or end of follow-up (30 June 2020). The risk time was split along an individual’s age in 1 year intervals using the ‘Lexis’ and ‘cutLexis’ functions in the ‘Epi’ package (version 2.47) [20] and ‘splitMulti’ function in the ‘popEpi’ package (version 0.4.10) in R version 4.0.2 (The R foundation for Statistical Computing, Vienna, Austria). Based on this time-split follow-up data, incidence rates of type 2 diabetes (outcome) as a function of age, sex and birthweight were modelled using Poisson regression, with current age included as a time-varying covariate. The age-specific incidence rate of type 2 diabetes was adjusted for relevant covariates in separate models using a complete case approach. Model 1 presents age-specific incidence rates for male and female participants and for levels of birthweight (continuous) and adjusted for prematurity status (term/preterm), parity and socioeconomic status. Model 2 was additionally adjusted for PS for birthweight and type 2 diabetes as well as maternal and paternal history of diabetes. Model 3 was additionally adjusted for adult BMI. The PS for type 2 diabetes and birthweight were standardised prior to analyses. Thus, the incidence rate ratios indicate the change in outcome per study population SD increase in PS for type 2 diabetes and birthweight.

Most participants were given a 2 h OGTT at the baseline examination. Thus, in sensitivity analyses, we further excluded people with a 2 h post OGTT glucose value ≥11.1 mmol/l (*n*=135) and followed the remaining individuals in the registers (*n*=3967).

All descriptive data are presented as mean (SD) for continuous variables and percentages for categorical variables. All analyses were done in R version 4.0.2.

### Ethics

The Inter99 baseline examination was approved by the Regional Scientific Ethics Committee in Denmark (KA 98 155) and registered at ClinicalTrials.gov (registration no. NCT00289237). The participants had provided written informed consent prior to inclusion. Access to and usage of the register data were approved by the Danish data protection authorities under the Capital Region of Denmark (P-2019–511).

## Results

### Characteristics of study participants

Table 1 presents selected background characteristics of the study population stratified by five birthweight categories. The total study population comprised 54% female participants; birthweight categories <3.5 kg contained a greater proportion of female participants and the birthweight categories ≥3.5 kg contained a greater proportion of male participants. At baseline, the mean age was 46.2 years, and differed across the birthweight categories, ranging from 45.2 to 47.4 years from the lowest to the highest birthweight category. The mean (SD) birthweight was 3.399 (0.524) kg, which is similar to the 2014 Danish child growth reference birthweight of 3.299 and 3.382 kg for boys and girls, respectively [21]. Ten per cent were born prematurely, and parity was positively related to birthweight. As expected, the PS for birthweight was higher in the higher birthweight categories, whereas we did not see any difference in PS for type 2 diabetes across the birthweight categories. Mean adult BMI increased from 25.8 kg/m^2^ to 26.7 kg/m^2^ in participants born with a birthweight of <2.5 kg and >4.0 kg, respectively.

**Table 1:** Characteristics at baseline (1999–2001) of the study population stratified by birthweight category

Characteristic	Birthweight						*p* value^a^	*n* missing
	Total	<2.5 kg	2.5–2.9 kg	3.0–3.4 kg	3.5–3.9 kg	>4.0 kg		
*N* analysed	4590	164	690	1616	1488	632		
Sex							<0.001	0
Male	46.0	34.1	37.1	42.6	50.1	57.8		
Female	54.0	65.9	62.9	57.4	49.9	42.2		
Age at baseline, years	46.2 (7.9)	45.2 (7.8)	45.3 (7.7)	45.8 (7.8)	46.7 (8.0)	47.4 (7.7)	<0.001	0
Birthweight, kg	3.399 (0.524)	2.119 (0.310)	2.767 (0.138)	3.225 (0.142)	3.674 (0.141)	4.220 (0.240)	<0.001	0
Prematurity status							<0.001	0
No	90.4	14.6	59.9	98.8	99.7	---		
Yes	9.6	85.4	40.1	1.2	0.3	---		
Parity							<0.001	51
Firstborn	35.7	48.4	47.6	40.1	31.2	18.7		
Second born	34.0	25.8	28.7	34.7	35.3	36.6		
Third or above	30.4	25.8	23.7	25.1	33.5	44.7		
PS of type 2 diabetes	52.64 (0.11)	52.66 (0.12)	52.65 (0.11)	52.64 (0.11)	52.64 (0.11)	52.64 (0.11)	0.190	154
PS of birthweight (multiplied by 1000)	26.88 (1.16)	26.69 (1.20)	26.66 (1.16)	26.75 (1.16)	27.00 (1.13)	27.21 (1.15)	<0.001	154
Paternal history of diabetes							0.383	0
No	92.3	89.6	93.3	91.6	92.7	92.6		
Yes	7.7	10.4	6.7	8.4	7.3	7.4		
Maternal history of diabetes							0.002	0
No	93.0	93.9	95.1	93.8	92.4	89.9		
Yes	7.0	6.1	4.9	6.2	7.6	10.1		
Socioeconomic status^b^							0.250	296
Not working, no education	2.7	4.7	4.0	2.8	2.1	2.0		
Not working, >1 year education	6.7	10.7	6.8	6.8	6.4	6.3		
Working, no education	11.9	10.0	12.4	11.9	11.6	12.7		
Working, >1 year education	78.6	74.7	76.8	78.4	79.9	79.0		
BMI, kg/m^2^	26.1 (4.5)	25.8 (4.7)	25.8 (4.4)	25.9 (4.5)	26.2 (4.4)	26.7 (5.0)	<0.001	<3

Data are presented as mean (SD) or as percentage for categorical variables. To avoid the display of microdata, --- is inserted where the whole number or percentage needs to be masked

^a^ Differences between strata were calculated by one-way ANOVA *F* test for continuous variables, Pearson’s χ^2^ test of independence for categorical variables with expected counts >4 in all cells, and Fisher’s exact test of independence for categorical variables with expected count in any cell <5

^b^ ‘Education’ refers to vocational, professional or academic adult education beyond primary or secondary school

### Association of birthweight with incidence of type 2 diabetes

**Fig. 2:**
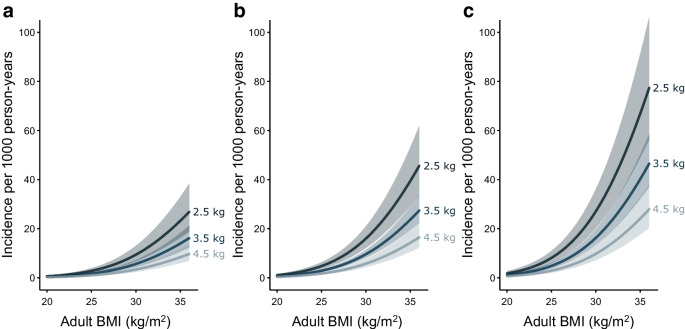
Incidence of type 2 diabetes per 1000 PY in male participants as a function of adult BMI, PS for type 2 diabetes, birthweight, sex, age, prematurity status, parity, PS for birthweight, maternal and paternal history of diabetes, and socioeconomic status (*n*=4102). The solid lines and shaded areas show the estimated incidence rates and 95% CIs, respectively, for male participants aged 55 years with adult BMI levels of 20–36 kg/m^2^, low (−1.5 SD) (**a**), average (0 SD) (**b**) and high (1.5 SD) (**c**) PS for type 2 diabetes and birthweight levels of 2.5, 3.5 and 4.5 kg, who were firstborns, who were term births, and who had a study population median PS for birthweight, no maternal and paternal history of diabetes and a high socioeconomic status (i.e. currently working with >1 year of education [referring to vocational, professional or academic adult education beyond primary or secondary school]). Age and BMI are specified as second-degree polynomials

**Fig. 3:**
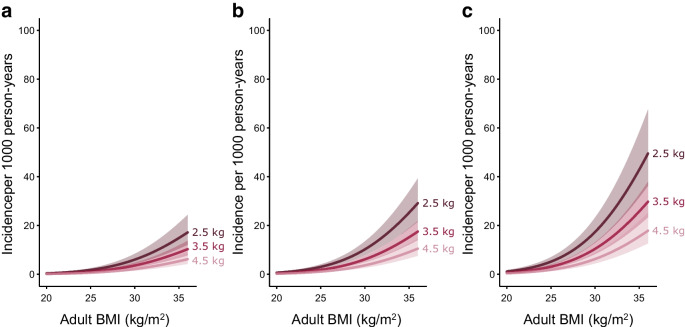
Incidence of type 2 diabetes per 1000 PY in female participants as a function of adult BMI, PS for type 2 diabetes, birthweight, sex, age, prematurity status, parity, PS score for birthweight, maternal and paternal history of diabetes, and socioeconomic status (*n*=4102). The solid lines and shaded areas show the estimated incidence rates and 95% CIs, respectively, for female participants aged 55 years with adult BMI levels of 20–36 kg/m^2^, low (−1.5 SD) (**a**), average (0 SD) (**b**) and high (1.5 SD) (**c**) PS for type 2 diabetes and birthweight levels of 2.5, 3.5 and 4.5 kg, who were firstborns, who were term births, and who had a study population median PS for birthweight, no maternal and paternal history of diabetes and a high socioeconomic status (i.e. currently working with >1 year of education [referring to vocational, professional or academic adult education beyond primary or secondary school]). Age and BMI are specified as second-degree polynomials

A total of 4590 participants were followed for 87,350.6 person-years (PY) (mean follow-up per person: 19 years). During follow-up there were 492 incident cases of type 2 diabetes (5.6 per 1000 PY). Birthweight was inversely associated with risk of type 2 diabetes (incidence rate ratio [95% CI] per 1 kg increase in birthweight in the fully adjusted model was 0.60 [0.48, 0.75]), with increasing incidence over age and higher risk among male participants (Table 2). The association of birthweight with risk of type 2 diabetes remained statistically significant across all models. The absolute rate of increase across age was higher in participants with a birthweight of 2.5 kg than in those with a birthweight of 3.5 kg and higher, independent of sex, prematurity status, parity, socioeconomic status, common genetic variation influencing birthweight and type 2 diabetes, maternal and paternal history of diabetes, and adult BMI (Fig. 1a, b). As there was no apparent interaction between age and birthweight, we assumed a multiplicative Poisson model. Thus, when presented data on the log-scale, showing the relative increase in type 2 diabetes incidence, the three predicted curves were parallel (ESM Fig. 2). Figures 2a–c and 3a–c show the estimated combined effects of birthweight, BMI and genetic risk of type 2 diabetes on type 2 diabetes incidence for the fully adjusted model 3, for male and female participants, and ESM Figs 3 and 4 show these data on the log-scale. For example, a 55-year-old man, born at term as firstborn, with a study population median PS for birthweight, no parental history of diabetes, highest socioeconomic status, a study population median PS for type 2 diabetes, a birthweight of 3.5 kg and a BMI of 25 kg/m^2^ has an estimated incidence rate of type 2 diabetes of 2.9 per 1000 PY. Keeping all things equal, if the birthweight was 2.5 kg (1 kg change equals 1.9 SD), the incidence rate was changed to 4.8 per 1000 PY. Instead, if the level of genetic susceptibility to type 2 diabetes was high (1.5 SD), the incidence rate of type 2 diabetes was changed to 4.9 per 1000 PY. However, if the level of BMI was 30 kg/m^2^ (5 kg/m^2^ equals 1.1 SD) or 35 kg/m^2^ (10 kg/m^2^ equals 2.2 SD), the incidence rate was changed to 9.6 and 23.7 per 1000 PY, respectively. If an obese male with BMI 35 kg/m^2^ and a study population median PS for type 2 diabetes has a birthweight of 2.5 kg instead of 3.5 kg, the estimated incidence rate of type 2 diabetes increases from 23.7 to 39.4 per 1000 PY. Further changing the genetic susceptibility to type 2 diabetes from average to high, the estimated incidence rate of type 2 diabetes was changed to 67.0 per 1000 PY. Interestingly, if the birthweight of this obese individual with high genetic susceptibility to type 2 diabetes is changed from 2.5 kg to normal (3.5 kg) or even high birthweight (4.5 kg), the estimated incidence rate of type 2 diabetes was changed to 40.2 (40% decrease) and 24.2 (64% decrease) per 1000 PY, respectively. Other factors from the multivariable Poisson regression associated with type 2 diabetes incidence rate included maternal and paternal history of diabetes and socioeconomic status (Table 2).

**Table 2:** Incidence rate ratios of type 2 diabetes during follow-up as a function of age, birthweight, sex and selected covariates

Variable	Model 1^a^	Model 2	Model 3	Model S
*N* analysed	4244	4104	4102	3967
Age, per year^b^	–	–	–	–
Birthweight, per kg	0.70 (0.57, 0.87)**	0.72 (0.58, 0.89)**	0.60 (0.48, 0.75)***	0.63 (0.49, 0.80)***
Sex				
Male	Ref.	Ref.	Ref.	Ref.
Female	0.63 (0.53, 0.77)***	0.62 (0.51, 0.75)***	0.64 (0.52, 0.78)***	0.75 (0.60, 0.93)*
Prematurity status				
No	Ref.	Ref.	Ref.	Ref.
Yes	0.93 (0.66, 1.32)	0.90 (0.62, 1.29)	0.75 (0.52, 1.09)	0.65 (0.42, 1.01)
Parity	0.99 (0.93, 1.06)	0.96 (0.89, 1.03)	0.98 (0.92, 1.05)	0.93 (0.86, 1.01)
Socioeconomic status^c^				
Not working, no education	Ref.	Ref.	Ref.	Ref.
Not working, >1 year education	0.81 (0.48, 1.39)	0.83 (0.48, 1.44)	0.89 (0.52, 1.55)	0.77 (0.42, 1.42)
Working, no education	0.63 (0.37, 1.06)	0.57 (0.34, 0.98)*	0.61 (0.36, 1.05)	0.50 (0.27, 0.91) *
Working, >1-year education	0.56 (0.35, 0.89) *	0.50 (0.31, 0.80) **	0.62 (0.38, 1.00)*	0.59 (0.35, 1.00)*
PS of type 2 diabetes, per SD		1.51 (1.37, 1.66)***	1.42 (1.29, 1.57)***	1.42 (1.28, 1.59)***
PS of birthweight, per SD		0.98 (0.89, 1.08)	0.98 (0.89, 1.07)	0.95 (0.86, 1.06)
Paternal history of diabetes				
No		Ref.	Ref.	Ref.
Yes		1.95 (1.49, 2.55)***	1.86 (1.42, 2.43)***	1.81 (1.33, 2.47)***
Maternal history of diabetes				
No		Ref.	Ref.	Ref.
Yes		1.96 (1.49, 2.57)***	1.63 (1.24, 2.15)***	1.71 (1.24, 2.34)***
BMI, per kg/m^2b^			–	–

^a^Model variables are estimated from Poisson regression and presented as incidence rate ratios with 95% CIs throughout. Models 1–3 are based on participants without diabetes at baseline, according to the Danish Diabetes Register, and model S is based on participants without type 2 diabetes at baseline, according to both the Danish Diabetes Register and the OGTT conducted at the baseline examination

^b^Risk estimates are not meaningful as the variables are specified as second-degree polynomials

^c^‘Education’ refers to vocational, professional or academic adult education beyond primary or secondary school

^*^p<0.05, **p<0.01, ***p<0.001

In sensitivity analysis, when further excluding people with type 2 diabetes according to their 2 h post OGTT glucose value, the overall findings did not change although, as expected, the CIs were a little wider (Table 2 [Model S] and ESM Fig. 5).

## Discussion

In a Danish population-based cohort of people aged 30–60 years at baseline in 1999–2001, we found that birthweight across the entire spectrum is inversely associated with the age- and sex-specific incidence rate of type 2 diabetes. The association was independent of other risk factors of type 2 diabetes (prematurity status, parity, parental history of diabetes, adult BMI and socioeconomic conditions), as well as known genetic factors influencing birthweight and type 2 diabetes as determined using genome-wide association studies (GWAS). The risk of developing type 2 diabetes increases strongly with age, and evidence of a higher age-related incidence of type 2 diabetes in people born with LBW is therefore important to better understand the role of developmental programming in the aetiology and pathophysiology of type 2 diabetes. Based on the Inter99 baseline examinations, we previously showed that birthweight was inversely associated with OGTT-assessed type 2 diabetes prevalence [14]. Uniquely, when excluding study participants who appeared with a diagnosis of either type 1 or 2 diabetes in the Danish Diabetes Register before the Inter99 baseline examination (main analysis) as well as further excluding participants with a 2 h OGTT-assessed type 2 diabetes diagnosis at baseline (sensitivity analysis), we consistently show an inverse association between birthweight and type 2 diabetes incidence over two decades. Accordingly, LBW is not only a risk factor for developing type 2 diabetes relatively early in life but also, even more so, as people become older.

While numerous studies published over the past three decades have established a clear link between birthweight and risk of developing type 2 diabetes, the more recent prospective study by Olaiya et al in American Indians showed an association between birthweight and an age-dependent risk of incident type 2 diabetes [22]. In this study, a U-shaped relationship between birthweight and type 2 diabetes risk was observed among participants aged 10–19 years, while an inverse linear association between birthweight and incident type 2 diabetes was observed in participants aged 20–39 years. The large differences in ages at type 2 diabetes onset in this study compared with our results are likely explained by ethnicity, illustrating the importance of studying the association between birthweight and age-dependent type 2 diabetes incidence rates in different populations and settings. However, ignoring the differences in overall risk and age at type 2 diabetes onset between the populations, our data are in general consistent with the results of Olaiya et al indicating an inverse association between birthweight and type 2 diabetes risk in adults.

Previous studies also including American Indians have suggested that the association of high birthweight with type 2 diabetes may reflect the well-known increased risk of type 2 diabetes associated with hyperglycaemia in pregnancy, including gestational diabetes [23-25]. Thus, the pathophysiological mechanisms underlying the association between LBW and type 2 diabetes are likely to differ significantly from those in people born with a high birthweight. Likewise, the clinical presentation of type 2 diabetes occurring due to either low or high birthweight may be different. For instance, the development of type 2 diabetes in people born with a high birthweight because of hyperglycaemia in pregnancy may, in contrast to type 2 diabetes associated with LBW, to some extent be mediated by an increased risk of early onset obesity [26]. The absence of an increased type 2 diabetes incidence rate at the high end of the birthweight spectrum in the current study could accordingly be explained by an earlier onset of type 2 diabetes in people with high as opposed to low birthweight and/or by adult obesity-mediated type 2 diabetes in people with a high birthweight. However, in the present analyses, we excluded participants with type 2 diabetes at the baseline examination and we saw a consistent inverse association of birthweight with type 2 diabetes incidence both before and after adjustment for adult BMI. It is therefore possible that a birthweight at the higher end of the spectrum constituted a beneficial phenotype in the 1940s to 1970s when the present study population was born. Before gestational diabetes was common, a high birthweight may not have involved very high adiposity and, therefore, building body mass during fetal life may in fact have improved metabolic capacity in the form of higher levels of lean mass, greater linear growth and to some extent also metabolically healthier fat mass. Our findings are supported by a study involving three large cohorts of men and women in the USA that also found the lowest diabetes risk in those with highest birthweight and lowest levels of unhealthy adult lifestyle [27]. Further studies are needed to understand why a high birthweight may either protect against or occur as a risk factor for type 2 diabetes in different populations.

Despite the overwhelming evidence from historical humanitarian famine catastrophes [9, 28] and experimental animal studies [11, 12] of a non-genetic origin of the association between LBW and risk of type 2 diabetes, the theoretical possibility of a quantitatively important degree of genetic confounding is still debated [29]. Indeed, data from carriers of glucokinase (MODY2) mutations provided proof of principle of the idea of genetic variants in the fetus influencing insulin secretion, causing fetal growth impairment and LBW [30]. Capitalising on GWAS discoveries over the past 15 years in a comprehensive aggregate genetic score [17], we were able to adjust the associations between birthweight and type 2 diabetes incidence rates for the known genetic contribution to type 2 diabetes. Furthermore, we also adjusted for birthweight PS and thus for known genetic contribution to birthweight variability. None of these adjustments attenuated the associations between birthweight and type 2 diabetes incidence. If anything, the adjustments had the opposite effect of strengthening the associations, underscoring the quantitative major contribution of non-genetic factors to the association between LBW and type 2 diabetes incidence. This is consistent with the fact that surprisingly few type 2 diabetes susceptibility genes influence birthweight and, conversely, that very few genes influencing birthweight are associated with risk of developing type 2 diabetes [18].

Although the absolute incidence rate of type 2 diabetes in this study was higher in male compared with female participants, we found no differences in the relative influence of birthweight on type 2 diabetes incidence when comparing the sexes (Fig. 1). In contrast, a previous Danish registry study by Zimmerman et al reported a stronger association between birthweight and type 2 diabetes risk in women compared with men [31]. Furthermore, this study also reported an increased risk of type 2 diabetes in the high birthweight spectrum among women but not among men. However, ascertainment of type 2 diabetes in the study by Zimmerman et al was determined from discharge diagnoses in hospital records and therefore did not include the vast proportion of individuals with type 2 diabetes in Denmark diagnosed in general practice. In contrast, type 2 diabetes in the current Inter99 cohort follow-up study was identified via the more recently established Danish Diabetes Registry and also included the relatively milder cases from general practice (ESM).

As illustrated in Figs 2a–c and 3a–c, the influence of birthweight on type 2 diabetes incidence was strongly dependent on BMI. Visualising the incidence rate of type 2 diabetes on a logarithmic scale as a function of age or BMI reveals curve linear relationships in which no indications appear of any excessive potentiating effects of either increasing age or BMI in male or female participants (ESM Figs 3, 4). In other words, the relative influence of age and BMI, respectively, on the association between birthweight and type 2 diabetes incidence rates appears similar across the entire birthweight spectrum. Similarly, when assessing the associations between birthweight and type 2 diabetes incidence in groups with low (−1.5 SD), median (0 SD) and high (1.5 SD) genetic susceptibility to type 2 diabetes, the relative associations within each group were found to be identical, while absolute incidence rate estimates for a given birthweight increases with increased genetic susceptibility. Taken together, these data indicate that the effects of birthweight, genetic susceptibility, BMI and, to some extent, age, are distinct and independent. Given the limitations of birthweight only representing a proxy of the fetal environment, BMI not necessarily capturing the totality of risk associated with excess adipose tissue accumulation and type 2 diabetes PS only accounting for the currently known type 2 diabetes genetic susceptibility, we are unable to determine the exact quantitative contributions made by each of these key type 2 diabetes risk factors with these data. Regardless, the quality and accuracy of estimates of these three distinct type 2 diabetes risk factors, along with the quality and data richness of the Inter99 cohort, collectively make the current data rather unique. As for the role and relative impact of the fetal environment vs genetics on type 2 diabetes incidence rate in the contemporary Danish population, our data clearly illustrate the individual contributions and relative importance of both factors, and further suggest that the contribution of fetal programming may be of similar importance to genetics in the aetiology of type 2 diabetes. To illustrate this, a person with a birthweight of 2.5 kg and a BMI of 35 kg/m^2^ within the lowest tertile of type 2 diabetes genetic susceptibility has approximately the same risk of developing type 2 diabetes as a person with the same BMI within the highest tertile of genetic type 2 diabetes susceptibility and a birthweight of 4.5 kg (Figs 2a–c and 3a–c).

Key strengths of this study include the long-term follow-up of age-specific incidence rates of type 2 diabetes in a representative population-based Danish cohort followed for more than two decades from a mean age of 46 years, representing the age window with highest incidence rates in European populations. People with type 2 diabetes according to the diabetes register were excluded at baseline. As a unique dimension assessing incidence rates of type 2 diabetes, we performed OGTT at the baseline examination, allowing us to further exclude individuals with OGTT-assessed type 2 diabetes at baseline, as a sensitivity analysis, and this did not change the overall findings. Further strengths include objectively measured weight at birth from original midwife records, extensive data on key covariates from both the Danish registers and the clinical examinations in the Inter99 study, as well as GWAS data in almost all participants, allowing estimations and adjustments for the currently known common genetic variation influencing birthweight and type 2 diabetes. Limitations included lack of accurate data on gestational age among the children born at term, as well as no objectively measured data on exposure to a hyperglycaemic intrauterine environment.

In conclusion, a lower birthweight is associated with a higher age-related incidence rate of type 2 diabetes through pathways independent of adult BMI, genetic susceptibility to type 2 diabetes and birthweight in an ageing Danish population, supporting the notion that the fetal environment influences risk of type 2 diabetes later in life, even in older adulthood. Future studies should examine how birthweight associates with other cardiometabolic outcomes and whether those who develop type 2 diabetes with a lower birthweight have a distinct cardiometabolic phenotype compared with those with a normal or high birthweight.

## Supplementary Information

Below is the link to the electronic supplementary material.

Supplementary file1 (PDF 1.41 MB)

## Data Availability

Data supporting the findings of this study are available from Statistics Denmark (https://www.dst.dk/en/TilSalg/Forskningsservice) and the Inter99 database (https://www.frederiksberghospital.dk/ckff/sektioner/sfe/bfu/inter99-20-%C3%A5r/Sider/default.aspx). Restrictions apply to the accessibility of these data, which were analysed under license for this study.

## Abbreviations

DOHaD: Developmental Origins of Health and Disease
GWAS: Genome-wide association studies
LBW: Low birthweight
PY: Person-years
PS: Polygenic score
