# Supplementary material for: Low birthweight is associated with a higher incidence of type 2 diabetes over two decades independent of adult BMI and genetic predisposition
Source: Diabetologia. 2023 Jun 12;66(9):1669–79. doi: 10.1007/s00125-023-05937-0 (PMC10390608; doi:10.1007/s00125-023-05937-0)
Supplement: Supplementary file 1 — Supplementary file1 (PDF 1.41 MB) [file 125_2023_5937_MOESM1_ESM.pdf]

Electronic supplementary material (ESM)

Table of contents

ESM text 2

Detailed description of the Danish Diabetes register 2

ESM figures 4

ESM references 9

ESM text

Detailed description of the Danish Diabetes register

A newly established Danish Diabetes Register (DMreg) provided information of type 2 diabetes (T2D) status and date of diagnosis. To increase the sensitivity for determining T2D status and date of diagnosis, DMreg was based on comprehensive data from the National Patient Register (NPR) (diagnosis of diabetes (ICD-8: 249, 250; ICD-10: E10, E11)) [1], the Medicines Products Register (MPR) (purchase of any anti-diabetic medication (ATC A10XXX)) [2], the National Health Service Registry (NHSR) (use of podiatry treatment for people with diabetes) [3], the Danish Adult Diabetes Database (DADD) (diagnosis of diabetes) [4], and the Eye Examination Database (Diabase) (diabetic eye examinations) [5]. The algorithm calculating diabetes status and date of diagnosis is described in detail elsewhere [6, 7]. In brief, the date of diabetes diagnosis was determined from the earliest available date in the NHSR, DADD, and the Diabase registers or the second of the first two date registrations in MPR or NPR. The first register in this prioritized order with available information on date of diagnosis was selected as the data source. For example, if information was not available for NHSR, DADD, Diabase, and a purchase of any anti-diabetic medication (e.g. insulin) was registered at two consecutive dates, the most recent of the two dates was selected as the data source. The second of the two purchase dates was used to avoid immortal time bias [6]. Subsequently, for persons with a registered date of diagnosis, the type of diabetes was determined. Based on the DADD register, a person was classified as type 1 if this diagnosis appeared in most of the records (e.g. 5 out of 8 available records), and similarly for T2D. If type 1 and 2 diabetes appeared equally or another type of diabetes was recorded as the main diagnosis, this person was left unclassified by the DADD. Subsequently, persons not registered or unclassified in the DADD were searched in NPR using a similar classification approach to the DADD. Finally, for persons not registered or unclassified in NPR, purchasing insulin before age 30, or oral anti-diabetic drugs before age 15, would classify them

as having type 1. Moreover, irrespective of the above criteria, persons without a recorded insulin purchase but a date of diagnosis were always classified as having type 2 [6].

ESM figures

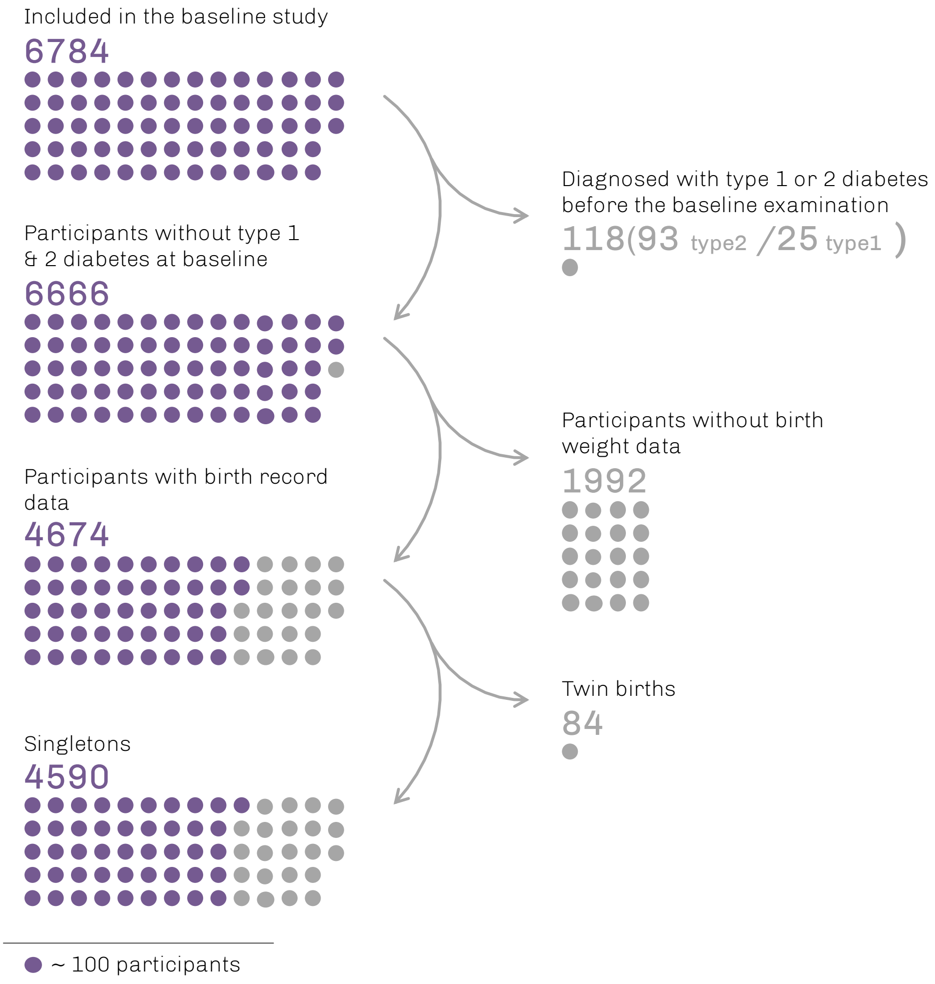

ESM Fig. 1 Participant flow.

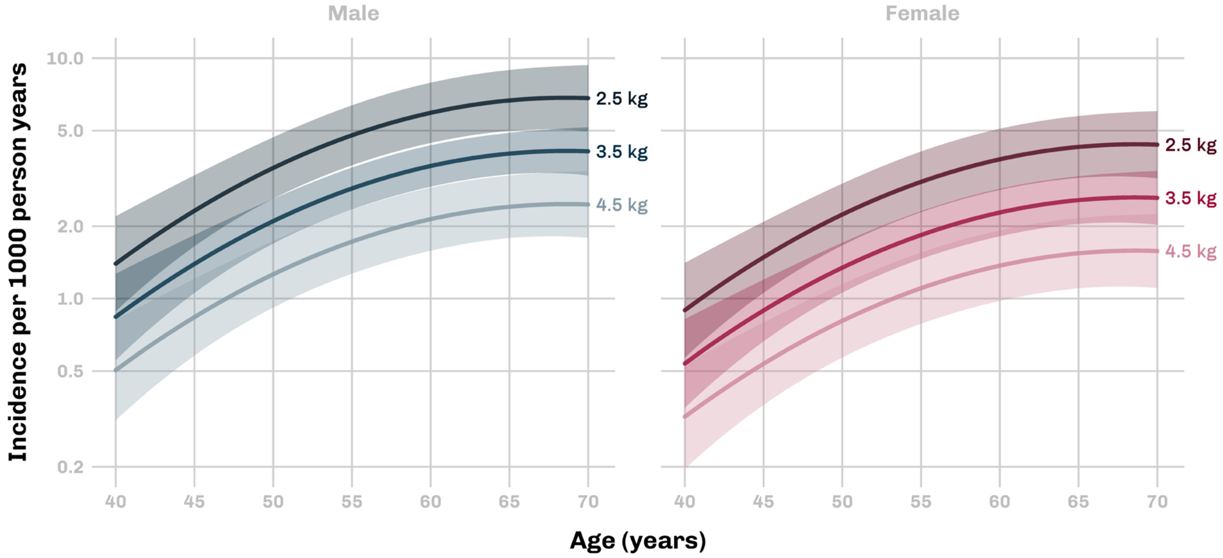

ESM Fig. 2 Incidence of type 2 diabetes per 1000 PY (on the logarithmic scale) as a function of age, birth weight, sex, prematurity status, parity, genetic risk score for birth weight and type 2 diabetes, maternal and paternal history of diabetes, socioeconomic status, and adult body mass index (BMI) (n = 4102). The solid lines and the shaded areas show the estimated incidence rates and 95% confidence limits, respectively, for participants aged 40–70 years, male and female participants, birth weight levels of 2.5, 3.5 and 4.5 kg, firstborns, term births, a study population median genetic risk score for birth weight and type 2 diabetes, no maternal- and paternal history of diabetes, a high socioeconomic status (i.e. currently working with >1 year of education [referring to vocational, professional or academic adult education beyond primary or secondary school]), and a body mass index (BMI) of 25kg/m². Age and BMI are specified as second-degree polynomials in the model.

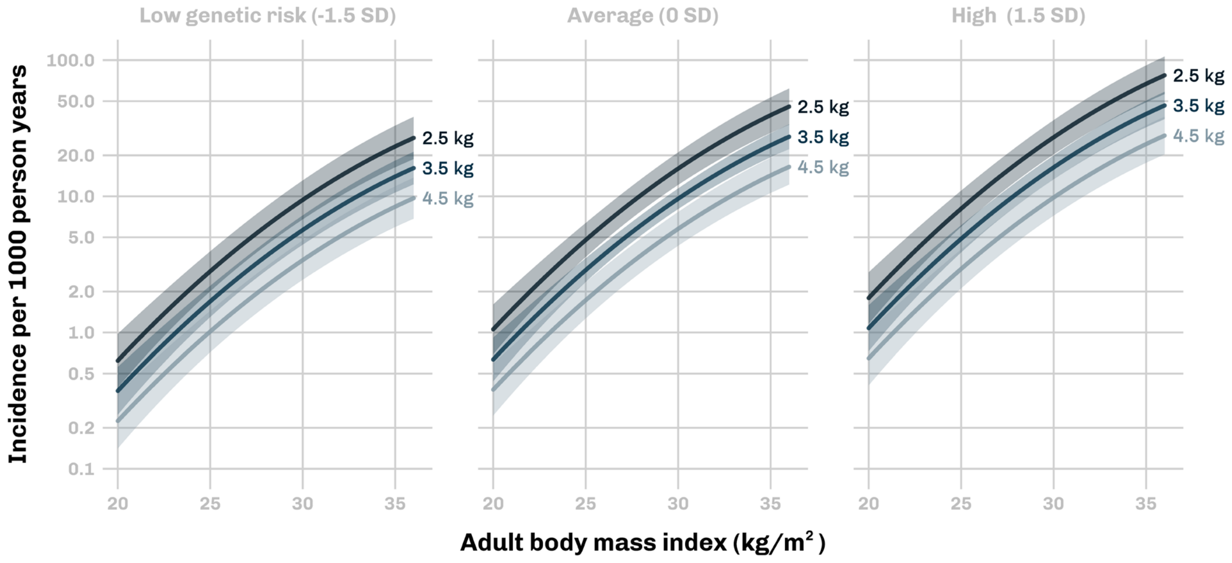

ESM Fig. 3 Incidence of type 2 diabetes per 1000 PY in male participants (on the logarithmic scale) as a function of adult body mass index, genetic risk score for type 2 diabetes, birth weight, sex, age, prematurity status, parity, genetic risk score for birth weight, maternal and paternal history of diabetes, and socioeconomic status (n = 4102). The solid lines and the shaded areas show the estimated incidence rates and 95% confidence limits, respectively, for male participants aged 55 years with adult BMI levels of 20–36 kg/m², low (-1.5 SD), average (0 SD), and high (1.5 SD) genetic risk scores for type 2 diabetes, birth weight levels of 2.5, 3.5 and 4.5 kg, firstborns, term births, a study population median genetic risk score for birth weight, no maternal- and paternal history of diabetes, a high socioeconomic status (i.e. currently working with >1 year of education [referring to vocational, professional or academic adult education beyond primary or secondary school]). Age and BMI are specified as second-degree polynomials.

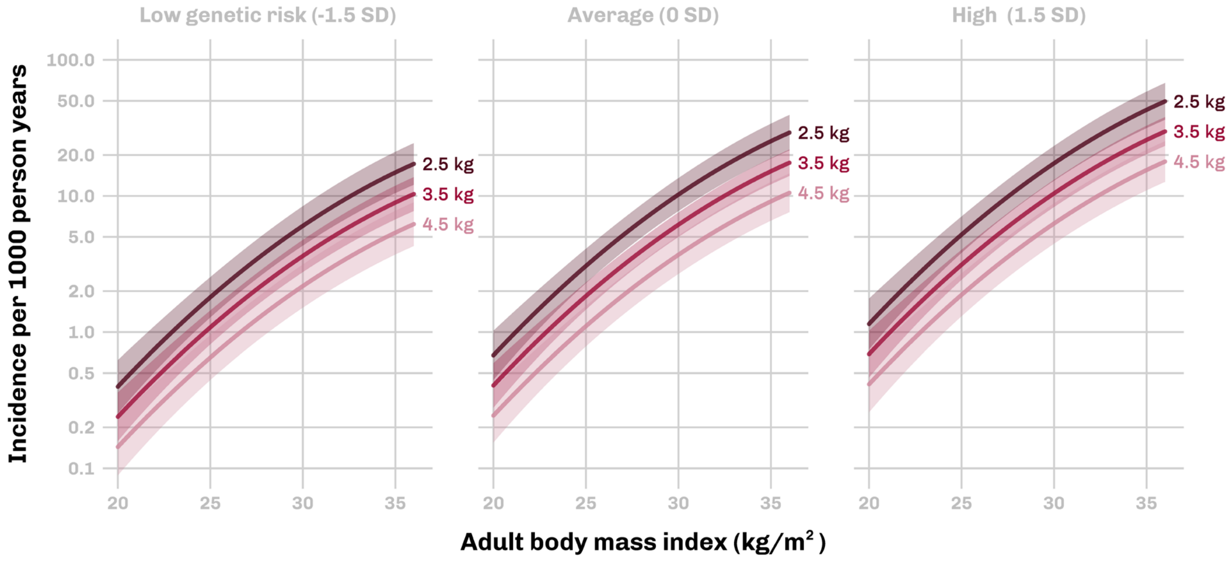

ESM Fig. 4 Incidence of type 2 diabetes per 1000 PY in female participants (on the logarithmic scale) as a function of adult body mass index, genetic risk score for type 2 diabetes, birth weight, sex, age, prematurity status, parity, gene risk score for birth weight, maternal and paternal history of diabetes, and socioeconomic status (n = 4102). The solid lines and the shaded areas show the estimated incidence rates and 95% confidence limits, respectively, for female participants aged 55 years with adult BMI levels of 20–36 kg/m², low (-1.5 SD), average (0 SD), and high (1.5 SD) genetic risk scores for type 2 diabetes, birth weight levels of 2.5, 3.5 and 4.5 kg, firstborns, term births, a study population median genetic risk score for birth weight, no maternal- and paternal history of diabetes, a high socioeconomic status (i.e. currently working with >1 year of education [referring to vocational, professional or academic adult education beyond primary or secondary school]). Age and BMI are specified as second-degree polynomials.

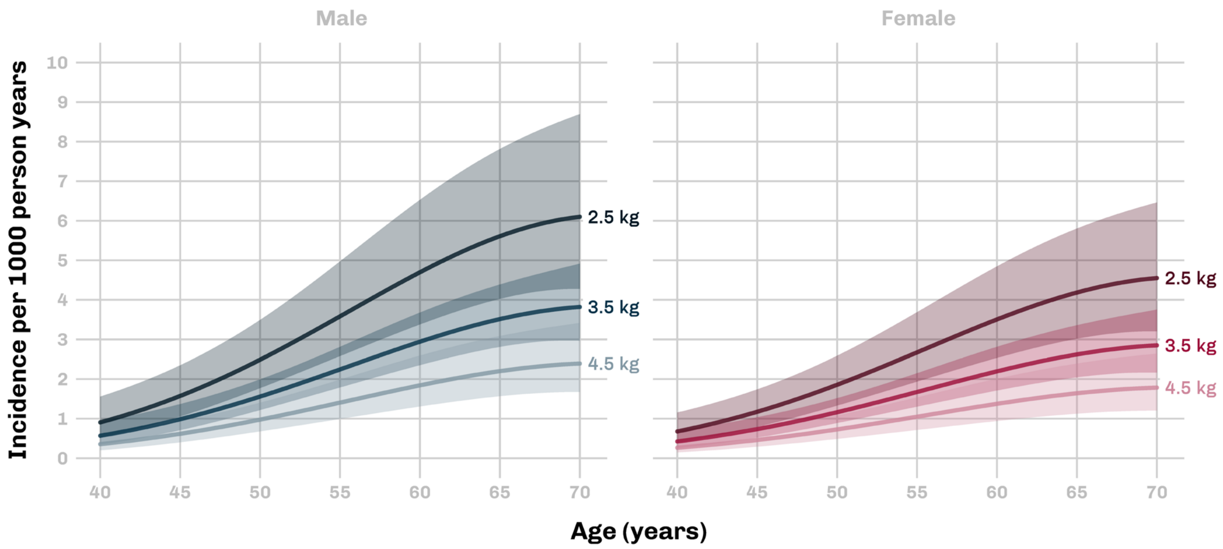

ESM Fig. 5 Incidence of type 2 diabetes per 1000 PY as a function of age, birth weight, sex, prematurity status, parity, genetic risk score for birth weight and type 2 diabetes, maternal and paternal history of diabetes, socioeconomic status, and adult body mass index (BMI) (n = 3967). The solid lines and the shaded areas show the estimated incidence rates and 95% confidence limits, respectively, for participants aged 40–70 years, male and female participants, birth weight levels of 2.5, 3.5 and 4.5 kg, firstborns, term births, a study population median genetic risk score for birth weight and type 2 diabetes, no maternal- and paternal history of diabetes, a high socioeconomic status (i.e. currently working with >1 year of education [referring to vocational, professional or academic adult education beyond primary or secondary school]), and a body mass index (BMI) of 25kg/m². Age and BMI are specified as second-degree polynomials in the model. This sensitivity analysis was restricted to persons without type 2 diabetes according to both the Danish Diabetes Register (DMreg) as well as the OGTT conducted at the baseline examination.

ESM references

[1] Lynge E, Sandegaard JL, Rebolj M (2011) The Danish National Patient Register. Scand J Public Health 39(7 Suppl): 30-33. 10.1177/1403494811401482

[2] Kildemoes HW, Sorensen HT, Hallas J (2011) The Danish National Prescription Registry. Scand J Public Health 39(7 Suppl): 38-41. 10.1177/1403494810394717

[3] Andersen JS, Olivarius Nde F, Krasnik A (2011) The Danish National Health Service Register. Scand J Public Health 39(7 Suppl): 34-37. 10.1177/1403494810394718

[4] Jorgensen ME, Kristensen JK, Reventlov Husted G, Cerqueira C, Rossing P (2016) The Danish Adult Diabetes Registry. Clin Epidemiol 8: 429-434. 10.2147/CLEP.S99518

[5] Andersen N, Hjortdal JO, Schielke KC, et al. (2016) The Danish Registry of Diabetic Retinopathy. Clin Epidemiol 8: 613-619. 10.2147/CLEP.S99507

[6] Carstensen B, Jørgensen ME (2019) A Danish Diabetes Register, http://bendixcarstensen.com/DMreg/Reg2016.pdf.

[7] Carstensen B, Ronn PF, Jorgensen ME (2020) Prevalence, incidence and mortality of type 1 and type 2 diabetes in Denmark 1996-2016. BMJ Open Diabetes Res Care 8(1). 10.1136/bmjdrc-2019-001071
